# Tell us what you really think: undergraduate nursing students validation of the Questionnaire Virtual Patient - a qualitative think-aloud study

**DOI:** 10.1186/s12909-026-09738-y

**Published:** 2026-06-23

**Authors:** Sophie Mårtensson, Joachim Eckerström, Rajna Knez, Karolina Sörman, Margaretha Larsson

**Affiliations:** 1 School of Health Sciences, University of Skövde, Högskolevägen 1, Skövde, 541 28 Sweden; 2 Department of Clinical Neuroscience, Centre for Psychiatry Research, Karolinska Institute & Stockholm Health Care Services, Stockholm, Region Stockholm Sweden; 3 Division of Nursing, Department of Neurobiology, Care Sciences and Society, Karolinska Institute, Huddinge, Sweden; 4 Skaraborg Hospital, Skövde, Sweden; 5 Institute of Neuroscience and Physiology, Sahlgrenska Academy, University of Gothenburg, Gothenburg, Sweden

**Keywords:** Cognitive validation, Digital healthcare education, Questionnaire, Think-aloud design, Undergraduate nursing students, Virtual patient simulation

## Abstract

**Background:**

The availability of digital technologies such as virtual patient simulation has nurtured innovation in higher healthcare education. However, the quality of validated questionnaires related to virtual patient simulation in higher healthcare education remains limited. In particular, there is a need for validated questionnaires for virtual patient simulations focusing on intimate partner violence. Thus, the aim was to cognitively validate questions in the Questionnaire Virtual Patient (QVP) among undergraduate nursing students.

**Method:**

A think-aloud design was employed. Eleven undergraduate nursing students from a single school of nursing in western Sweden participated in an individual digital cognitive interview. Collected data were analyzed using a qualitative conventional analysis. The QVP consists of eleven background questions and additional eighteen questions, structured into two themes: (1) *User friendliness virtual patient* and (2) *Virtual patient as an integrated part of educational module.*

**Result:**

The QVP was perceived as clear, well-constructed, and easy to understand. At the same time, some areas for refinement were emphasized. Thus, original questions and revised versions of the questions in QVP are presented in the alignment with the structure of the QVP.

**Conclusion:**

The cognitive validation of the QVP highlights the need for methodological rigor and thoughtful planning in survey design. Ongoing refinement of questionnaires can contribute to the development of evidence-based learning didactics in an increasingly digital healthcare educational context.

**Supplementary Information:**

The online version contains supplementary material available at 10.1186/s12909-026-09738-y.

## Background

The availability of digital technologies has nurtured innovation in higher education [1], leading educators to adopt more constructive approaches to healthcare education [2, 3]. Today, educators increasingly assume the role of learning facilitators rather than traditional lecturers, recognizing students as active learners capable of attributing individual meanings to their personal experiences and construct their own knowledge over time [4, 5]. This aligns with Malcolm Knowles’ principles of andragogy, which emphasize that adult learners engage more actively when content is relevant to their needs, when they have a role in shaping their learning process and assessment, and when new knowledge builds upon prior experiences [6]. With thoughtful planning, that promotes an active learning environment based on challenges and clearly defined learning objectives that fosters deep learning by prioritizing understanding and the application of knowledge over memorization and recall [7, 8], digital and virtual simulation using virtual patients is an instrument to meet each of these principles.

Virtual simulation recreates authentic clinical situations using virtual patient displayed on a computer screen; thus, active learning is necessitating from the student as communication skills, decision-making and psychomotor skills is practiced [9]. Virtual simulation with virtual patients enhances interaction and feedback and increases both the perception of self-efficacy and student satisfaction level [7, 10]. Moreover, the use of virtual simulation with virtual patient in healthcare educations improves the development of competencies necessary to practice patient safety [11, 12].

One complex clinical area requiring the development of competencies necessary to practice patient safety is intimate partner violence. The World health Organization [13] refers to intimate partner violence as “behavior by an intimate partner or ex-partner that causes physical, sexual or psychological harm, including physical aggression, sexual coercion, psychological abuse and controlling behaviors”. Exposure to intimate partner violence is associated with a wide range of serious short and long-term physical, psychological, sexual and reproductive health consequences [14, 15].

Despite healthcare providers unique position to identify and respond to intimate partner violence, they often encounter significant barriers, including limited education on respect and empathy, a lack of training on how to ask questions and how to act on the responses, unclear guidelines and procedures, time constraints, and persistent gender inequalities and norms on the acceptability [16–18]. Given the profound and lasting impact of intimate partner violence, along with the challenges healthcare providers encounter, it is essential to integrate intimate partner violence specific learning objectives, anchored in clearly defined knowledge objectives into higher healthcare education curricula [19].

To facilitate this integration, best practice aligned educational interventions are needed [20]. Allison et al. [21], reported that healthcare educators employ a variety of learning didactics to facilitate learning about intimate partner violence; including theoretical lectures, a variety of simulation instruments and seminars. Further, they highlighted the importance of subject matter expertise and emphasized digital learning didactics, particularly virtual simulations involving virtual patients, as a valuable and necessary instrument for developing intimate partner violence related competencies.

However, Sereno et al. [22], identified significant variations in the design and implementation of intimate partner violence related questionnaires in their systematic review of empirical studies. This inconsistency hindered the identification of best practice strategies. Similarly, several review studies focusing on virtual simulation with virtual patient in healthcare education have found that the quality of validated questionnaires remains inadequate [23, 24]. For example, Kononowicz et al. [25], in their systematic review and meta-analysis, reported that most of the questionnaires used across 51 studies lacked proper validation. Sharma and Ruijkar [26] emphasize that validation represent the most critical pashes of survey-based research, as several factors determine the overall effectiveness of a survey. These include not only the design and language quality of the questions asked but also the ability of the survey to meaningfully engage participants.

To address this gap, we employed a think-aloud design to cognitively validate questions in a newly designed Questionnaire Virtual Patient (QVP). In educational research, the think-aloud design provides valuable insights for the cognitive validation [27], as it captures participants’ perceptions and thought processes while they engage in completing a questionnaire [28, 29]. Based on this, the aim was to cognitively validate questions in the QVP among undergraduate nursing students.

## Materials and methods

### Study design used

This study employed a think-aloud design [27, 28] to cognitively validate the QVP, which forms part of a larger umbrella project investigating undergraduate nursing students’ experiences of using a virtual patient as part of an educational module focused on intimate partner violence [12, 30, 31]. Data were collected through individual digital cognitive interviews [32], with eleven undergraduate nursing students from a single school of nursing in western Sweden. The transcribed interview data were analyzed using a qualitative conventional analysis with an inductive approach as outlined by Hsieh and Shannon [33].

### Participants

Participants were undergraduate nursing students enrolled in the fourth semester of a six-semester Bachelor of Science in Nursing program at a school of nursing in western Sweden. In Sweden, the nursing degree is regulated by the Higher Education Ordinance (1993:100) and comprises 180 ECTS-credits (European Credit Transfer and Accumulation System), equivalent to three years of full-time study [34]. Of these 180 credits, a mandatory knowledge objective is to demonstrate knowledge of men’s violence against women and violence in intimate relationships.

On the curricula course-site all undergraduate nursing students were informed by an independent teacher who had no active role in the course or study design; that they had the opportunity to participate in the study and, that participation was voluntary, confidential and that they could withdraw from the study at any time without impact of their course grade. In this way, informed consent was subsequently obtained from eleven students, nine females and two males, ranging in age from 25 to 44 years (M = 28), all of whom voluntarily agreed to participate in an individual digital cognitive interview [31], conducted by the first author, in alignment with previous think-aloud design [27, 35]. Although Nielsen [36] suggest that 77–85% of usability issues can be identified within five interviews, a larger sample size was selected in this study to ensure data richness and thematic saturation.

### The QVP

The QVP questionnaire was developed to evaluate an educational module on intimate partner violence, with a focus on the use of a virtual patient. The QVP, which encompasses both quantitative and qualitative insights [37] was developed by a cross-disciplinary research team which included co-authors. In addition, co-authors also provided some input on the QVP prior to its implementation in the studies conducted at the school of nursing [12, 31]. The design of the QVP facilitated the collection of standardized, comparable measures inspired by the Technology Acceptance Model [38] and the Unified Theory of Acceptance and Use of Technology [39] focusing on aspects such as user-friendliness, clarity of instructions and overall experiences using virtual simulation with virtual patient. In addition, open-ended questions were included to investigate participants experiences on the strengths and limitations of the educational module focusing intimate partner violence, as well as reflect on how these skills can be applied in clinical practice. The QVP comprises eleven background questions, including gender, age, prior work experiences in healthcare, and two themes: (1) *User-friendliness virtual patient* and (2) V*irtual patient as an integrated part of educational module.* The first theme includes twelve questions rated using both Likert-scale and slider-scales, accompanied by nine opportunities for participants to elaborate on their responses in free-text. The second theme consists of six questions rated on a Likert-scale, each with an option for further development through free-text responses. The interactive educational module encompassed three parts: a digital lecture, a virtual patient, and a dialog-based seminar, as described in a previous studies [12, 31]. Original questions from the first and second themes, along with revised versions, are presented in the result section. The finalized version of the QVP, including background questions and revised questions from both themes, is provided in the Appendix.

### Procedure

Data collection for the study was conducted during the winter of 2024/2025. Prior to data collection, the individual digital cognitive interviews [32], conducted by first author were piloted and validated through discussions with co-author, who has expertise in the think-aloud design. Participants were invited by first author to take part in an individual digital cognitive interview. At the beginning of each interview, participants were presented with instructions adapted from Van Someren et al. [40], along with the QVP. Any questions raised by participants were addressed by first author at this stage. Once participants began working through the QVP, they were not interrupted unless they remained silent for approximately ten seconds, at which point they were encouraged to continue thinking aloud. Each interview was assigned a unique code to ensure anonymity, and no personally identifiable information was collected. Interviews lasted between 10 and 27 min (M = 14 min), were audio recorded, and transcribed verbatim by a professional transcriber.

### Analysis

The interview data were aggregated for analysis using a qualitative conventional analysis with an inductive approach as outlined by Hsieh and Shannon [33]. Qualitative conventional analysis seeks to describe a phenomenon in a condensed manner by identifying how different codes relate and cluster together [33]. Initially, each transcribed interview was compiled into a single document and read through multiple times to achieve immersion and obtain a sense of the whole. This initial interpretation was discussed among the authors involved in the analysis, to ensure a shared understanding of the data. Once a sense of the whole was established, the data were read word by word to derive codes related to the study’s aim. These codes were further condensed into simplified statements and organized under each question within the two themes of the QVP. To enhance trustworthiness [41], first author reviewed the condensed codes with co-authors, until consensus was reached. Finally, each question was subsequently revised by first author in alignment with all authors. The analysis was iterative, involving continuous movement between the whole text and its parts.

### Result

The result of cognitive validation of each question (Q) and revised question (RQ) is presented in alignment with the structure of the QVP two themes:

1. *User-friendliness virtual patient*
2. *Virtual patient as an integrated part of educational module*

### User-friendliness virtual patient

Q1) *How do you perceive your digital competence in general*, i.e.,* how accustomed are you to digital meetings and digital platforms?* was perceived by the students as clearly formulated. However, several participants noted that the term *digital competence* is inherently broad and can encompass a wide range of digital environments, including websites, databases, and social media platforms; each of which may require distinct skills and forms of knowledge. As one student expressed:

“It concerns how one understands digital technology; for instance, how one navigates and works within digital platforms, the extent to which they are used, and the degree of familiarity or learning acquired.”

This suggests that students interpret digital competence not as a singular ability, but as a composite of experiences and proficiencies across various digital technological contexts. Consequently, RQ1) *How do you perceive your digital competence in general*, i.e.,* how accustomed are you to digital meetings and digital platforms (e.g.*,* digital meetings*,* platforms*,* websites*,* databases*,* or social media)?* was formulated.

Q2) *Do you have previous experience of training with a virtual patient?* was generally perceived by the majority of students as clear, concrete, and easy to understand, with little risk of being misinterpreted. One student succinctly interpreted the question as:

“That is, if I have previously trained with a virtual patient.”

However, some students suggested that the question could be further refined by specifying the type of virtual patient being referred to. For instance, they noted that virtual patients may vary significantly in format, including generative AI-based simulations, pre-recorded text interactions, or recorded video scenarios. Thus, RQ2) *Have you had any previous experience with virtual patient simulation? (e.g.*,* AI-driven simulations*,* interactive text-based cases*,* or pre-recorded video scenarios)?* was framed.

Q3) *How did you experience navigating the VP platform?* was perceived by the majority of students as unclear. Specifically, many students indicated that the abbreviation *VP* was unfamiliar and not immediately recognizable as referring to a virtual patient. *As* one student expressed:

“Here I need to think about whether the concept “VP”’ maybe means virtual patient, it’s not certain that everyone will understand it.”

Students generally recommended that the abbreviation be written out in full to avoid confusion. In addition, some students found the term navigate ambiguous, expressing uncertainty about how it should be interpreted in this context. One student commented:

“I don’t know what it means to navigate on VP, or what the VP platform is, does it refer to the virtual patient or the platform where we trained with the virtual patient?”

These quotations suggest that both the terminology and phrasing of the question may benefit from clarification to ensure consistent understanding among respondents. Accordingly, RQ3) *How did you experience the process of navigating the virtual patient platform?* was developed.

Q4) *How did you experience the instructions on the VP platform?* was perceived by students as short and concise. However, similar to the previous question, most students noted that the abbreviation VP, presumably referring to virtual patient was not a familiar term. Consequently, RQ4) *How did you experience the instructions provided on the virtual patient platform?* was formulated.

Q5) *Do you think the dialogue questions that were available to choose from were sufficiently concrete and varied?* was perceived by students as clear. They felt that the wording was specific enough to convey that the question was asking for their personal perceptions and interpretations of the dialogue options provided. As one student stated:

“Yes, clear question. Similarly, you understand the meaning of it, or it is good to have bold, you understand what the question is about, and yes, I understand the context.”

However, a few students indicated that the question could benefit from further clarification, as it was not immediately clear which dialogue questions were being referred. Additionally, some students remarked that the question felt somewhat repetitive, as it echoed the focus of the two preceding questions. Thus, RQ5) *Were the dialogue options available during the virtual patient simulation clear*,* and varied enough to support your decision-making?* was framed.

Q6) *How did you experience the scope—was there a reasonable number of questions to choose from?* was perceived by the vast majority of students as clear and easy to understand. However, a few students found the term scope to be ambiguous and required additional reflection to interpret its meaning. These students expressed uncertainty about what scope referred to, whether it pertained to the overall number of questions within the platform or specifically to the questions directed at the virtual patient. One student expressed:

“Is it the scope of questions in the platform or the scope of questions to the virtual patient?”

To improve clarity, some students suggested replacing the term scope altogether, with one recommending the word options as a more intuitive alternative. Consequently, RQ6) *How did you experience the number of questions available; did you feel there were enough options to choose from during the virtual patient simulation?* was formulated.

Q7) *How did you perceive the logic of the feedback from the patient?* was considered clear by the vast majority of students. One student interpreted it as referring to the realism of the virtual patient’s responses, stating:

“I perceive the question as asking about the logic of the feedback from the virtual patient; I interpret it as how realistic the feedback was.”

However, a few students found the term logic to be ambiguous and questioned its necessity in the question. These students suggested that removing the word might enhance clarity, as its inclusion could introduce confusion regarding the intended focus of the question. So, RQ7) *How did you perceive the feedback from the virtual patient? Did it seem realistic and appropriate to the situation?* was framed.

Q8) *How did you perceive the logic of the feedback from the expert?* was generally perceived by students as clear. Similar to the previous question concerning feedback from the virtual patient, students understood the focus to be on the coherence and realism of the responses, though in this case, the feedback was attributed to the expert. Thus, RQ8) *How did you perceive the feedback from the expert? Did it seem realistic and relevant to your performance?* was formulated.

Q9) *How would you rate your experience of using virtual patients? (On a scale from 1 to 10*,* where 1 represents ‘bad’ and 10 represents ‘very good’)* was perceived by students as clear and easy to understand. They recognized that the question was specifically aimed at evaluating their overall experience with virtual patients. Several students appreciated the explicit explanation of the scale, noting that it enhanced the clarity of the response options. As one student stated:

“Using a scale is a good way to get a number on how I experienced it.”

However, some students also expressed reservations about using a numerical scale, noting its subjective nature. One student reflected:

“I have a bit of difficulty with those 1–10 scales—a ten for me may not be a ten for someone else, or I may never dare; I sometimes don’t dare to put a ten even though I may think it is great.”

This feedback suggests that while the question is mostly effective and clearly formulated, there may be inherent limitations in using fixed numerical scales to capture subjective experiences. Consequently, RQ9) *How would you rate your overall experience using virtual patients on a scale from 1 to 10*,* where 1 represents ‘unsatisfactory´ and 10 represents ‘very satisfactory´? was formulated*.

Q10) *How interested are you in using virtual patients again during your training? (On a scale of 1 to 10*,* where 1 represents ‘never’ and 10 represents ‘definitely’)* was perceived by students as clear. The vast majority compared it to the previous question, acknowledging that the use of a numerical scale with clearly defined endpoints (1 as *never* and 10 as *definitely*) facilitated understanding. However, some students noted that this question felt more like a binary or yes/no inquiry compared to the earlier one. Consequently, a few students suggested that employing a 1-to-10 scale might be unnecessary in this context and that a simpler response format could be sufficient. Accordingly, RQ10) *How interested are you in using virtual patients again during your training? Please rate on a scale from 1 to 10*,* where 1 means ‘not at all interested’ and 10 means ‘very interested.* was developed.

Q11) *In your opinion*,* what are the advantages of using virtual patients in your education?* was generally perceived by students as clear and accessible, effectively inviting both positive and negative reflections. One student highlighted that the phrase *in your opinion* was beneficial, as it explicitly framed the question as seeking personal perspectives. The use of straightforward language and the full-term virtual patient, rather than an abbreviation were also appreciated. Several students valued the opportunity to provide free-text responses, noting that this allowed for more nuanced and individualized answers, which they felt had been lacking in previous questions. However, one student remarked on the potential difficulty of answering such open-ended questions, expressing a concern that responses might become somewhat performative:

“You want to answer what you think they want to hear, like ‘this is great,’ and it can feel a bit contrived, as if I have to come up with something impressive.”

This highlights the challenge of eliciting authentic feedback in open-ended survey questions. So, RQ11) *In your opinion*,* what are the main advantages or challenges of using virtual patients in your education?* was formulated.

Q12) *In your opinion*,* what are the disadvantages of using virtual patients in your training?* was perceived by students as clear and easy to understand. Many noted its similarity to the preceding question about advantages, appreciating that it similarly invited personal reflection and reasoning. However, in this question, students recognized that the focus was on eliciting their own critical or negative perspectives regarding training with virtual patients. Thus, RQ12) *In your opinion*,* what are the main disadvantages of using virtual patients in your education?* was framed.

### Virtual patient as an integrated part of educational module

Q13) *How relevant did you find the digital lecture on intimate partner violence that you received before the VP?* was perceived by students as clear and well-formulated. However, some students noted an inconsistency in the use of the abbreviation *VP*, which they found potentially confusing. One student appreciated that the phrase *digital lecture* was highlighted, as it helped emphasize the focus of the question. At the same time, a few students suggested that the question could be improved by specifying which digital lecture was being referred to, as the training included multiple segments. As one student reflected:

“I had to think about it there; it was like training with a bit of mixed segments. So maybe just specify ‘digital training.”

These responses indicate that while the question is largely effective, further clarification would enhance precision and reduce ambiguity. Consequently, RQ13) *How relevant did you find the digital lecture on intimate partner violence that you received before the virtual patient simulation?* was formulated.

Q14) *Did you experience the training with the virtual patient as beneficial for your learning about intimate partner violence?* was perceived by students as clear and well-posed. Many found it easy to interpret, particularly in terms of whether the virtual patient contributed to their understanding of intimate partner violence. One student remarked:

“It was one of those questions where I immediately thought of an answer like this, yes, I didn’t even need the answer options. But I still think it’s good to have several options because it encourages more responses. Otherwise, it’s just yes or no, and this is clearer. The question was asked so simply that you immediately got an answer in your head.”

Consistent with previous feedback, students appreciated that the phrase virtual patient was written out in full rather than abbreviated, which enhanced clarity. So, RQ14) *Did you experience the training with the virtual patient as beneficial for your learning about intimate partner violence?* was not reframed.

Q15) *How valuable was the dialogue-based seminar to your learning about intimate partner violence?* elicited mixed responses from students. While some perceived the question as clearly stated, others found it ambiguous, particularly regarding the meaning of dialogue-based seminar. One student reflected:

“Yes, but that question was perhaps a bit vague. Dialogue-based seminar, did you mean when we sat in a small group and discussed? Or was it the one with the video? Or when we watched the films? Or when we talked in a group at school?”

This uncertainty suggests a need for clearer specification of the seminar format. Additionally, several students noted a grammatical issue, pointing out the omission of the preposition for before *your* in the question. One student commented:

“Maybe it wasn’t very clear, yes, probably because of the grammar.”

These insights indicate that both conceptual clarity and grammatical precision are important for ensuring unambiguous comprehension of the question. Thus, RQ15) *How valuable was the dialogue-based seminar (i.e.*,* the small-group discussion following the virtual patient simulation) for your learning about intimate partner violence?* was formulated.

Q16) *How well do you think the different learning elements (digital lecture*,* virtual patient*,* and dialogue-based seminar) were integrated and complemented each other?* was described by students as clear and easy to understand. They emphasized that explicitly naming the learning didactics contributed to the clarity of the question by providing necessary context. Consequently, RQ16) *How well do you think the different learning didactics*,* digital lecture*,* virtual patient*,* and dialogue-based seminar*,* were integrated and supported each other in enhancing your learning?* was framed.

Q17) *Which part of the following did you find most valuable for your learning about intimate partner violence?* was perceived by students as well-constructed and easy to understand. One student suggested a minor improvement, stating:

“What you could have done was to line up all these again, but otherwise, you’re just over the moon if you were to get the wording perfect. The question itself is easy to understand.”

This response indicates that while the question is effective, presenting the response options more clearly or sequentially could enhance its usability. Accordingly, RQ17) *Which of the following learning didactics digital lecture*,* virtual patient*,* and dialogue-based seminar did you find most valuable for your learning about intimate partner violence?* was developed.

Q18) *In what way do you think the combination of digital lectures*,* training with VP*,* and seminars will benefit your clinical work in the future?* was generally perceived by students as clear and easy to understand. They appreciated that the question invited reflection on specific learning didactics relevant to their experience. One student articulated this well, stating:

“It asks which elements you are interested in, how it has affected me, and how it can benefit me moving forward in my clinical work.”

However, a few students suggested that the term clinical work could be further clarified to avoid ambiguity. Additionally, some students noted the repeated use of the abbreviation VP and expressed a preference for consistently writing out virtual patient or ensuring the abbreviation is clearly defined. As one student commented:

“If you want to use the abbreviation, it’s good to have it explained at the beginning, but otherwise, it’s easy enough to write out.”

These insights highlight the importance of consistent terminology and clear phrasing to support respondent comprehension. Consequently, RQ18) *In what way do you think the combination of digital lectures*,* training with the virtual patient*,* and dialogue-based seminars will benefit your future clinical practice or work as a nurse?* was formulated.

## Discussion

The present study employed a think-aloud design [27, 28] to cognitive validate the two themes of the QVP: *(1) User-friendliness of the virtual patient* and *(2) Virtual patient as an integrated part of the educational module.* As Charters [42] notes, the think-aloud design is closely connected to Vygotsky’s [43] seminal work *Thought and Language* and his concept of *inner speech*. Vygotsky theorized that the transition from thought to speech passes through a stage of meaning before being expressed in words [43].

Expressed in words, the undergraduate nursing students generally perceived the two themes of the QVP as clear, well-constructed, and easy to understand. This perception aligns with the theoretical foundation of the QVP, which draw inspiration from the Technology Acceptance Model [37] and the Unified Theory of Acceptance and Use of Technology [38]. Yet, the result reflects nuanced perception of inconsistency in the interpretation of technological terminology. For example, the terminology related to digital competence, navigation and instructions elicited uncertainties, mainly due to lack of specificity regarding the technological context. Furthermore, the result clearly showed that the term virtual patient or its abbreviation VP has been used inconsistently. These results support Kishore’s [44] assertion that, without careful planning, survey design and development can be challenging and suboptimal. Thus, the result reinforces the importance of thorough planning in survey design and development, emphasizing the need for critical reflection on question content, particularly regarding the intended meaning of each question. As illustrated in the Appendix, refining technological terminology into more user-friendliness language and avoiding unexplained abbreviations can substantially enhance clarity and promote more consistent interpretation among respondents, an essential factor in contexts where accuracy in data collection is critical. This is consistent with Artino [45] who emphasizes that a well-constructed survey strengthens the overall validity and reliability of collected data.

Drawing up on this, the result underscores that the majority of the questions within the two themes of the QVP were well-constructed, effectively using Likert-scale and slider-scales formats. At the same time, the result provides a nuanced picture of the inherent subjectivity associated with such scaled response formats. For example, the hesitation in assigning high numerical values despite positive experiences, highlighting the limitations of quantitative scaling methods: the inability to fully capture the complexity and subtlety of individual experiences and perceptions [32]. Thus, a notable methodological strength of question design within the two themes of the QVP lies in the inclusion of open-ended questions. The result displayed that the open-ended questions created opportunities to express one’s own words, allowing for deeper expressive reflections. However, the result also revealed the implicit pressure to provide socially desirable or acceptable responses, which introduces the potential for response bias. Overall, these results are consistent with the broader criticism of qualitative methodology design. Sharma [26] emphasizes that multiple methodological design factors influence the overall quality of questionnaires. In light of this, the result strengthens the need for methodological rigor and thoughtful planning in survey design and development, especially in the context of evaluating complex higher healthcare educational interventions, such as assessing undergraduate nursing students’ learning experiences using virtual patient simulation.

This was evidenced in the results by the QVP questions addressing a variety of learning didactics (e.g., digital lecture, virtual patient, and dialogue-based seminar) which were not only perceived as a well-constructed but also as facilitating reflective practice that enabled deeper learning about intimate partner violence. As Dewey [46] emphasizes, reflective practice is not merely a rational intellectual process but an active process of deriving meaning from experience. This aligns with Malcolm Knowles’ principles of andragogy, which emphasize that adult learners engage more effectively when new knowledge builds up on prior experiences [6]. With thoughtful planning, well-constructed questionnaires such as the QVP can serve purposes other than ensuring methodological rigor. The overall positive perceptions of the QVP reinforces the result in Eckerström [12] and Mårtensson [31] both of which focus on virtual patient simulations addressing intimate partner violence in higher healthcare education, where data collection was conducted using the QVP. As Philiphs [47] stresses, the think-aloud design can serve as a valuable instrument for evaluating the clarity of questionnaires currently employed in the field.

However, some limitations should be acknowledged. First, the transferability of the study result is limited by the use of a think-aloud design [27, 28], thus one cannot necessarily generalize think-aloud data from one population to another [47], and a qualitative conventional analysis with an inductive approach as outlined by Hsieh and Shannon [33]. Second, the study was conducted with a relatively small, convenience sample of undergraduate nursing students from a single school of nursing in western Sweden, which may limit the generalizability of the findings beyond this specific context. Moreover, all students enrolled in the curricula course-site were informed about the opportunity to participate by an independent teacher who had no involvement in the course or study design, helping to reduce coercion. However, the subset of students who volunteered to participate may have had a greater interest in developing the subject than those who did not volunteer, providing a selection bias. Future studies could address this limitation by employing multi-site recruitment strategies at the national or international level to capture a broader and more diverse range of educational contexts, thus enhancing the external validity of the results. Lastly, while the study focused specifically on how undergraduate nursing students perceive the questions in the QVP, it did not assess the actual learning outcomes associated with the use of virtual patient simulation. Future studies should aim to evaluate the educational impact of virtual patient simulation, particularly in terms of their effectiveness in achieving intended learning outcomes when integrated into broader curricular modules.

## Conclusion

In conclusion, the think-aloud validation of the QVP provide valuable insights into how undergraduate nursing students perceive survey questions related to *User-friendliness of the virtual patient* and *Virtual patient as an integrated part of the educational module*. This study also helps to address a gap in the existing literature by contributing to the limited body of knowledge on validated questionnaires for virtual patient simulation focusing on intimate partner violence. Generally, the QVP were perceived as a clear, well-constructed, and easy to understand. At the same time, areas for refinement in the result are presented. Addressing this result reinforces the importance of methodological rigor and careful planning in survey design and development. Continued refinement of questionnaires can support the development of evidence-based learning didactics in an increasingly digital healthcare educational environment.

## Supplementary Information

Supplementary Material 1.

Supplementary Material 2.

## Data Availability

The data is available upon reasonable request. To request access to the underlying research data, please contact the corresponding author.

## Abbreviations

QVP: Questionnaire Virtual Patient
