# Supplementary material for: Tell us what you really think: undergraduate nursing students validation of the Questionnaire Virtual Patient - a qualitative think-aloud study
Source: BMC Med Educ. 2026 Jun 23;26:1022. doi: 10.1186/s12909-026-09738-y (PMC13289443; doi:10.1186/s12909-026-09738-y)
Supplement: Supplementary file 1 — Supplementary Material 1. [file 12909_2026_9738_MOESM1_ESM.docx]

**The Questionnaire Virtual Patient (QVP)**

**Background questions**

**Sex:** Men ☐ Female ☐ Don’t want to give up ☐

**Age:** 18–25 ☐ 26–30 ☐ 31–35 ☐ 36–40 ☐ 41–45 ☐ 50–55 ☐ 56–60 ☐ 61—65 ☐ >65 ☐

**At which university are you enrolled in a healthcare education program while participating in the virtual patient simulation?** ______________________________________________________________________________________________________________________________________________________________________________________________________________________________________________________________

**Which semester are you currently enrolled in within your healthcare education?** ______________________________________________________________________________________________________________________________________________________________________________________________________________________________________________________________

**Do you have any clinical experience working in healthcare?**  Yes ☐ No ☐

**If yes, in which area(s)?** ______________________________________________________________________________________________________________________________________________________________________________________________________________________________________________________________

**If yes, how many years of clinical experience do you have working in healthcare?**

< 1 ☐ 1–3 ☐ 4–7 ☐ 8–10 ☐ >10 ☐

**If yes, do you have experience asking patients about intimate partner violence?** Yes ☐ No ☐

**Have you ever chosen not to ask about intimate partner violence even though you believed it may have been necessary?** Yes ☐ No ☐

**If yes, what do you believe were the reasons for not asking about intimate partner violence?** _____________________________________________________________________________________________________________________________________________________________________________________________________________________________________________________________________________________________________________________________________________________________________________________________

**Theme 1: User-friendliness virtual patient**

The section corresponding to theme 1 presents questions related to the user-friendliness of the virtual patient.

1. **How do you perceive your digital competence in general, i.e., how accustomed are you to digital meetings and digital platforms (e.g., digital meetings, platforms, websites, databases, or social media)?**
   High competence ☐ Medium competence ☐ Low competence ☐ Very low competence ☐
2. **Have you had any previous experience with virtual patient simulation? (e.g., AI-driven simulations, interactive text-based cases, or pre-recorded video scenarios)?**Yes ☐ No ☐
3. **How did you experience the process of navigating the virtual patient platform?**Very easy to navigate ☐ Quite easy to navigate ☐ Quite difficult to navigate ☐ Very difficult to navigate ☐
4. **How did you experience the instructions provided on the virtual patient platform?**Very easy to understand ☐ Quite easy to understand ☐ Quite difficult to understand ☐ Very difficult to understand ☐
5. **Were the dialogue options available during the virtual patient simulation clear, and varied enough to support your decision-making?**Yes ☐ No ☐
   Free text response: _____________________________________________________________________________________________________________________________________________________________________________________________________________________________________________________________________________________________________________________________________________________________________________________________
6. **How did you experience the number of questions available; did you feel there were enough options to choose from during the virtual patient simulation?**Yes ☐ No ☐
   Free text response: _____________________________________________________________________________________________________________________________________________________________________________________________________________________________________________________________________________________________________________________________________________________________________________________________
7. **How did you perceive the feedback from the virtual patient? Did it seem realistic and appropriate to the situation?**Very easy to understand ☐ Quite easy to understand ☐ Quite difficult to understand ☐ Very difficult to understand ☐
   Free text response: _____________________________________________________________________________________________________________________________________________________________________________________________________________________________________________________________________________________________________________________________________________________________________________________________
8. **How did you perceive the feedback from the expert? Did it seem realistic and relevant to your** **performance?**Very easy to understand ☐ Quite easy to understand ☐ Quite difficult to understand ☐ Very difficult to understand ☐
   Free text response: _____________________________________________________________________________________________________________________________________________________________________________________________________________________________________________________________________________________________________________________________________________________________________________________________
9. **How would you rate your overall experience using virtual patients on a scale from 1 to 10, where 1 represents ‘very poor’ and 10 represents ‘very good’?**1 ☐ 2 ☐ 3 ☐ 4 ☐ 5 ☐ 6 ☐ 7 ☐ 8 ☐ 9 ☐ 10 ☐
   Free text response: _____________________________________________________________________________________________________________________________________________________________________________________________________________________________________________________________________________________________________________________________________________________________________________________________
10. **How interested are you in using virtual patients again during your training? Please rate on a scale from 1 to 10, where 1 means ‘not at all interested’ and 10 means ‘very interested.**1 ☐ 2 ☐ 3 ☐ 4 ☐ 5 ☐ 6 ☐ 7 ☐ 8 ☐ 9 ☐ 10 ☐
    Free text response: _____________________________________________________________________________________________________________________________________________________________________________________________________________________________________________________________________________________________________________________________________________________________________________________________
11. **In your opinion, what are the main advantages or challenges of using virtual patients in your education?**Free text response: _____________________________________________________________________________________________________________________________________________________________________________________________________________________________________________________________________________________________________________________________________________________________________________________________
12. **In your opinion, what are the main disadvantages or challenges of using virtual patients in your education?**Free text response: _____________________________________________________________________________________________________________________________________________________________________________________________________________________________________________________________________________________________________________________________________________________________________________________________

**Theme 2: Virtual patient as an integrated part of educational module**

The section corresponding to theme 2 presents questions related to the virtual patient as an integrated part of educational module.

1. **How relevant did you find the digital lecture on intimate partner violence that you received before the virtual patient simulation?**Very relevant ☐ Quite relevant ☐ Not very relevant ☐ Not at all relevant ☐
   Free text response: _____________________________________________________________________________________________________________________________________________________________________________________________________________________________________________________________________________________________________________________________________________________________________________________________
2. ***Did you experience the training with the virtual patient as beneficial for your learning about intimate partner violence?***Very relevant ☐ Quite relevant ☐ Not very relevant ☐ Not at all relevant ☐
   Free text response: _____________________________________________________________________________________________________________________________________________________________________________________________________________________________________________________________________________________________________________________________________________________________________________________________
3. **How valuable was the dialogue-based seminar (i.e., the small-group discussion following the virtual patient simulation) for your learning about intimate partner violence?**Very relevant ☐ Quite relevant ☐ Not very relevant ☐ Not at all relevant ☐
   Free text response: _____________________________________________________________________________________________________________________________________________________________________________________________________________________________________________________________________________________________________________________________________________________________________________________________
4. **How well do you think the different learning didactics, digital lecture, virtual patient, and dialogue-based seminar, were integrated and supported each other in enhancing your learning?**Very integrated ☐ Quite integrated ☐ Not very integrated ☐ Not at all integrated ☐
   Free text response: _____________________________________________________________________________________________________________________________________________________________________________________________________________________________________________________________________________________________________________________________________________________________________________________________
5. **Which of the following learning didactics digital lecture, virtual patient, and dialogue-based seminar did you find most valuable for your learning about intimate partner violence?**Digital lecture ☐ Virtual patient ☐ Dialogue-based seminar ☐
   Free text response: _____________________________________________________________________________________________________________________________________________________________________________________________________________________________________________________________________________________________________________________________________________________________________________________________
6. **In what way do you think the combination of digital lectures, training with the virtual patient, and dialogue-based seminars will benefit your future clinical practice or work as a nurse?**Free text response: _____________________________________________________________________________________________________________________________________________________________________________________________________________________________________________________________________________________________________________________________________________________________________________________________
