# Supplementary material for: Tell us what you really think: undergraduate nursing students validation of the Questionnaire Virtual Patient - a qualitative think-aloud study
Source: BMC Med Educ. 2026 Jun 23;26:1022. doi: 10.1186/s12909-026-09738-y (PMC13289443; doi:10.1186/s12909-026-09738-y)
Supplement: Supplementary file 2 — Supplementary Material 2. [file 12909_2026_9738_MOESM2_ESM.docx]

**Virtuell Patient Enkät**

**Bakgrundsfrågor**

**Kön:** Man ☐ Kvinna ☐ Vill ej uppge ☐

**Ålder:** 18–25 ☐ 26–30 ☐ 31–35 ☐ 36–40 ☐ 41–45 ☐ 50–55 ☐ 56–60 ☐ 61—65 ☐ >65 ☐

**Vid vilket universitet studerar du när du deltog i virtuell patient simulering?** ______________________________________________________________________________________________________________________________________________________________________________________________________________________________________________________________

**Vilken termin läser du?** ______________________________________________________________________________________________________________________________________________________________________________________________________________________________________________________________

**Har du klinisk erfarenhet av att arbeta inom vården?**  Ja ☐ Nej ☐

**Om ja, vilken/vilka områden?** ______________________________________________________________________________________________________________________________________________________________________________________________________________________________________________________________

**Om ja, hur många år av klinisk erfarenhet har du av att arbeta inom vården?**

< 1 ☐ 1–3 ☐ 4–7 ☐ 8–10 ☐ >10 ☐

**Om ja, har du erfarenhet av att fråga patienter om våld i nära relation?** Ja ☐ Nej ☐

**Har du valt att inte fråga om våld i nära relation även när du upplevt det nödvändigt?** Ja ☐ Nej ☐

**Om ja, vad tror du var orsaken till att inte fråga om våld I nära relation?** _____________________________________________________________________________________________________________________________________________________________________________________________________________________________________________________________________________________________________________________________________________________________________________________________

**Tema 1: Användarvänlighet virtuell patient**

Tema 1 presenterar frågor relaterade till den virtuella patientens användarvänlighet.

1. **Hur uppfattar du din digitala kompetens generellt? dvs. hur van är du vid digitala möten och digitala plattformar (tex., webbplatser, databaser eller sociala medier)?**

Hög kompetens ☐ Medelhög kompetens ☐ Låg kompetens ☐ Väldigt låg kompetens ☐

1. **Har du tidigare erfarenhet av att simulera med virtuell patient (tex. AI-driven, interaktiv textbaserad eller för inspelad video virtuell patient)?**Ja ☐ Nej ☐
2. **Hur uppfattade du det att navigera på den virtuella patient plattformen?**Väldigt lätt att navigera ☐ Ganska lätt att navigera ☐ Ganska svårt att navigera ☐ Väldigt svårt att navigera ☐
3. **Hur uppfattade du instruktionerna på den virtuella patient plattformen?**Väldigt lätt att navigera ☐ Ganska lätt att navigera ☐ Ganska svårt att navigera ☐ Väldigt svårt att navigera ☐
4. **Uppfattade du att dialogfrågorna under den virtuella patienten simuleringen var tydliga och varierande nog för att stödja ditt beslutsfattande?**

Ja ☐ Nej ☐
Fri text: _____________________________________________________________________________________________________________________________________________________________________________________________________________________________________________________________________________________________________________________________________________________________________________________________

1. **Hur uppfattade du feedbacken från den virtuella patienten? Var den realistisk och ändamålsenlig till situationen?**Väldigt lätt att uppfatta ☐ Ganska lätt att uppfatta ☐ Ganska svårt att uppfatta ☐ Väldigt svårt att uppfatta ☐
   Fri text: _____________________________________________________________________________________________________________________________________________________________________________________________________________________________________________________________________________________________________________________________________________________________________________________________
2. **Hur uppfattade du feedbacken från experten? Var den realistisk och ändamålsenlig till situationen?**Väldigt lätt att uppfatta ☐ Ganska lätt att uppfatta ☐ Ganska svårt att uppfatta ☐ Väldigt svårt att uppfatta ☐
   Fri text: _____________________________________________________________________________________________________________________________________________________________________________________________________________________________________________________________________________________________________________________________________________________________________________________________
3. **Hur skulle du skatta din generella upplevelse av att simulera med virtuell patient på en skala från 1 – 10, där 1 representerar ’väldigt låg’ och 10 ’väldigt bra’?**1 ☐ 2 ☐ 3 ☐ 4 ☐ 5 ☐ 6 ☐ 7 ☐ 8 ☐ 9 ☐ 10 ☐
   Fri text: _____________________________________________________________________________________________________________________________________________________________________________________________________________________________________________________________________________________________________________________________________________________________________________________________
4. **Hur skulle du skatta ditt intresse av att simulera med virtuell patient under din utbildning på en skala från 1 – 10, där 1 representerar ’inte alls intresserad’ och 10 ’väldig intresserad’?**1 ☐ 2 ☐ 3 ☐ 4 ☐ 5 ☐ 6 ☐ 7 ☐ 8 ☐ 9 ☐ 10 ☐
   Fri text: _____________________________________________________________________________________________________________________________________________________________________________________________________________________________________________________________________________________________________________________________________________________________________________________________
5. **Enligt dig, vad är fördelarna med virtuell patient simulering i utbildning?**Fri text: _____________________________________________________________________________________________________________________________________________________________________________________________________________________________________________________________________________________________________________________________________________________________________________________________
6. **Enligt dig, vad är nackdelarna med virtuell patient simulering i utbildning?**Fri text: _____________________________________________________________________________________________________________________________________________________________________________________________________________________________________________________________________________________________________________________________________________________________________________________________

**Tema 2: Virtuell patient som en integrerad del av lärmodul**

Tema 2 presenterar frågor relaterade till virtuell patient som en integrerad del av lärmodul.

1. **Hur relevant uppfattade du att den digitala föreläsningen innan simuleringen med virtuell patient var för ditt lärande om våld i nära relation?**Väldigt relevant ☐ Ganska relevant ☐ Inte särskilt relevant ☐ Inte alls relevant ☐
   Fri text: _____________________________________________________________________________________________________________________________________________________________________________________________________________________________________________________________________________________________________________________________________________________________________________________________
2. **Hur relevant uppfattade du att simuleringen med virtuell patient var för ditt lärande om våld i nära relation*?***Väldigt relevant ☐ Ganska relevant ☐ Inte särskilt relevant ☐ Inte alls relevant ☐
   Fri text: _____________________________________________________________________________________________________________________________________________________________________________________________________________________________________________________________________________________________________________________________________________________________________________________________
3. **Hur relevant uppfattade du att dialogseminariet dvs. seminariet som följde efter simuleringen med virtuell patient var för ditt lärande om våld i nära relation?**Väldigt relevant ☐ Ganska relevant ☐ Inte särskilt relevant ☐ Inte alls relevant ☐
   Fri text: _____________________________________________________________________________________________________________________________________________________________________________________________________________________________________________________________________________________________________________________________________________________________________________________________
4. **Uppfattade du att de olika didaktikerna, dvs. digital föreläsning, virtuell patient simulering och dialogseminarium var integrerade med varandra för att stödja ditt lärande om våld i nära relation?**

Väldigt integrerade ☐ Ganska integrerade ☐ Inte särskilt integrerade ☐ Inte alls integrerade ☐
Fri text: _____________________________________________________________________________________________________________________________________________________________________________________________________________________________________________________________________________________________________________________________________________________________________________________________

1. **Vilken av de följande didaktikerna, dvs. digital föreläsning, virtuell patient simulering och dialogseminarium uppfattade du bidrog mest till ditt lärande om våld i nära relation?**Digital föreläsning ☐ Virtuell patient ☐ Dialogseminarium ☐
   Fri text: _____________________________________________________________________________________________________________________________________________________________________________________________________________________________________________________________________________________________________________________________________________________________________________________________
2. **Hur tror du kombinationen av digital föreläsning, virtuell patient simulering och dialogseminarium om våld i nära relation kan stödja ditt framtida kliniska arbete?**Fri text: _____________________________________________________________________________________________________________________________________________________________________________________________________________________________________________________________________________________________________________________________________________________________________________________________
